# IgE Regulates the Expression of smMLCK in Human Airway Smooth Muscle Cells

**DOI:** 10.1371/journal.pone.0093946

**Published:** 2014-04-10

**Authors:** Jyoti Balhara, Naresh Singh Redhu, Lianyu Shan, Abdelilah S. Gounni

**Affiliations:** Department of Immunology, Faculty of Medicine, University of Manitoba, Winnipeg, Manitoba; Ludwig-Maximilians-University Munich, Germany

## Abstract

Previous studies have shown that enhanced accumulation of contractile proteins such as smooth muscle myosin light chain kinase (smMLCK) plays a major role in human airway smooth muscle cells (HASM) cell hypercontractility and hypertrophy. Furthermore, serum IgE levels play an important role in smooth muscle hyperreactivity. However, the effect of IgE on smMLCK expression has not been investigated. In this study, we demonstrate that IgE increases the expression of smMLCK at mRNA and protein levels. This effect was inhibited significantly with neutralizing abs directed against FcεRI but not with anti-FcεRII/CD23. Furthermore, Syk knock down and pharmacological inhibition of mitogen activated protein kinases (MAPK) (ERK1/2, p38, and JNK) and phosphatidylinositol 3-kinase (PI3K) significantly diminished the IgE-mediated upregulation of smMLCK expression in HASM cells. Taken together, our data suggest a role of IgE in regulating smMLCK in HASM cells. Therefore, targeting the FcεRI activation on HASM cells may offer a novel approach in controlling the bronchomotor tone in allergic asthma.

## Introduction

Asthma is a chronic inflammatory disease of the airways, clinically characterized by airway obstruction, inflammation, and hyperresponsiveness. Inflammatory components of this disease include an increased infiltration of activated T lymphocytes, mast cells, eosinophils, and neutrophils within the airway lumen and bronchial submucosa [1], [2]. Besides inflammatory cells, airway smooth muscle (ASM) cells also play an important role in the development of asthma. Human ASM (HASM) cells are primary effector cells that control the contractile aparatus within the airways [3].

It is well recognized that most asthma in children and adults is associated with atopy, characterized by an increased synthesis of IgE against common allergens. Indeed, two-thirds of asthmatics are allergic and more than 50% of patients with severe asthma have allergy [4]. Bronchial hyperresponsiveness was shown to be associated with serum IgE levels [5], and transferable by IgE-rich serum from asthmatic to non-asthmatic individuals [6]. Furthermore, serum IgE levels play an important role in smooth muscle hyperreactivity [5], [7], [8] and incubation of IgE-rich serum from atopic individuals causes hyperreactivity in isolated airway preparations from non atopic patients [9]. Moreover, IgE was proposed to induce smooth muscle contractile function through binding to the smooth muscle membrane and cause subsequent hyperpolarization [10]. Contractility of ASM cells is principally controlled by the activity of smooth muscle isoform (130 kDa) of myosin light chain kinase (smMLCK), predominantly expressed in HASM cells [11], [12]. Initiation of contraction involves the activation of calcium-calmodulin complex which activates smMLCK and subsequently phosphorylates 20 kDa myosin regulatory light chain and triggers contraction [13]. smMLCK content has been shown to be increased in atopic sensitized human [14], and ragweed-sensitized canine airway smooth muscle [15] and is associated with enhanced contractility in bronchus passively sensitized with serum [14] and HASM cells from asthmatic subjects [16]. Collectively, although serum IgE is thought to affect ASM phenotype and function, there is little evidence of a direct role of IgE in modulating smMLCK expression.

Previously, we and others have shown that HASM cells express the high and low affinity IgE receptor (FcεRI) and (FcεRII/CD23)[17], [18]. FcεRI expression is highly regulated [19] in HASM cells, which may explain the difficulty of detection, experienced by Xia *et al* [20], that we have discussed recently [21], [22]. FcεRI cross-linking on HASM cells induces the release of proinflamatory cytokines and chemokines and led to transient increase in intracellular calcium (Ca^2+^) mobilization [17]. Moreover, FcεRI expression in HASM cells is regulated by proinflammatory and Th-2 cytokines [19]. More recently, we have reported that IgE induces the expression of pro-allergic thymic stromal lymphopoietin (TSLP) in a spleen tyrosine kinase (Syk) and NF-κB-dependent manner [23]. Taken together, IgE has been demonstrated to induce multiple pro-inflammatory mediator release in HASM cells that may participate in airway inflammatory response. In this study, we report that IgE can upregulate smMLCK expression in HASM cell through FcεRI activation. IgE-induced smMLCK upregulation involves Syk, mitogen activated protein kinase (MAPK) (ERK1/2, P38, and JNK), and PI3K signaling elements.

## Materials and Methods

### Ethics Statement

All the experimental procedures were approved by the Human Research Ethics Board of the University of Manitoba, Winnipeg, MB, Canada. Written informed consent for HASM harvesting was obtained from all patients.

### Reagents

Purified human IgE (monoclonal, non-immune; azide free) was obtained from Antibody shop (BioPorto Diagnostics A/S, Denmark). Fetal bovine serum (FBS) and sodium pyruvate were purchased from HyClone (Logan, UT, USA). 100X L-glutamine, DMEM, Ham’s F-12, trypsin-EDTA, and antibiotics (penicillin, streptomycin) were purchased from Invitrogen Canada Inc. (Burlington, ON, Canada). All other reagents were purchased from Sigma unless specified. Monoclonal anti Human FcεRI α chain (mAb15-1) was kindly provided by Dr. Franz Kriceck, (Novartis Research Institute,Vienna, Austria). Anti CD23/FcεRII mAb (clone M-L233) was purchased from BD biosciences.

### Preparation of Human Airway Smooth Muscle (HASM) Cells

HASM cells were obtained from macroscopically healthy segments of second to fourth generation lobar or main bronchus of patients undergoing surgery for lung adenocarcinoma as we described previously [24], [25]. To extend the life span of these cells, primary low-passage cultures were infected with a retrovirus vector encoding the (hTERT) gene. The expression of hTERT was verified in immortalized cells by RT-PCR using telomerase-specific primers. Immortalized cells were passaged (4∶1 dilution) up to 50 times with no evidence of senescence [24], [26]. Furthermore, hTERT HASM cells at confluence retain smooth muscle-specific actin, SM22, and calponin protein expression and mobilize intracellular Ca^2+^ in response to acetylcholine, a physiologically relevant contractile agonist [24]. As shown in Figure S1A, serum starvation induces the arrest of cells in G1/M phase (propidium Iodide staining) and enhanced expression of calponin in these cells as a smooth muscle maturation and differentiation marker [24] (Figure S1B).

### Cell Stimulation

Sub-confluent HASM cells were serum deprived for 48 h in Ham’s F-12 medium containing 5 μg/ml human recombinant insulin, 5 μg/ml human transferrin, 5 ng/ml selenium, and antibiotics (100 U/ml penicillin and 100 μg/ml streptomycin). Cells were then stimulated with IgE (1, 5 and 10 μg/ml) in fresh FBS-free medium for the specified time. We found similar preliminary observations with all doses tested; therefore we performed experiments with 5 μg/ml. In neutralizing experiments, cells were pre-treated with mouse anti-human FcεRI (IgG1, clone mAb15-1), mouse anti-human CD23/FcεRII (M-L233), or mouse IgG1 isotype control (MOPC21) (all at 10 μg/ml) for 1 hr prior to IgE stimulation. In some experiments, cells were also pretreated with Erk1/2 inhibitor U-0126 (10 μM), P38 inhibitor SB-203580 (10 μM), JNK inhibitor SP600125 (40 nM) or phosphatidylinositol-3-kinase (PI3K) inhibitor Wortmannin (100nM) for 45 minutes prior to stimulation with IgE.

### RNA Isolation and Real-time RT-PCR Analysis

Serum-deprived confluent HASM cell cultures were stimulated for the specified time, harvested, and total cellular RNA was extracted using TRIzol method (Invitrogen Canada Inc., Burlington, ON). Reverse transcription was performed by using 2 μg of total RNA in a first-strand cDNA synthesis reaction with High Capacity cDNA Reverse transcriptase kit as recommended by the supplier (Applied Biosystems, Foster City, CA, USA. Primers for human housekeeping gene, glyceraldhyde-3-phosphate dehydrogenase (GAPDH) are forward primer 5′-AGCAATGCCTCCTGCACCACCAAC-3′ and reverse primer 5′-CCGGAGGGGCCATCCACAGTCT-3′. Primers for smMLCK are forward Primer: 5′ GACTGCAAGATTGAAGGATAC 3′ and Reverse Primer: 5′ GTTTCCACAATGAGCTCTGC 3′. Real-time quantitative PCR was carried out using ABI 7500 Real-Time PCR System and analyzed by 7500 System SDS software version 1.3.1 (Applied Biosystems, Foster City, CA, USA), following manufacturer’s instructions. Product specificity was determined by melting curve analysis and by visualization of PCR products on agarose gels. Calculation of the relative amount of each cDNA species was performed according to standard protocols. Briefly, the amplification of smMLCK gene in stimulated cells was calculated first as the copy number ratio of smMLCK to GAPDH, and then expressed as normalized values of fold increase over the value obtained with unstimulated (control) cells.

### Western Blot

For western blots, HASM cells were lysed for 2 min on ice in M-PER lysis buffer (Thermo Scientific) supplemented with a cocktail of protease inhibitors (Sigma-Aldrich) and centrifuged for 20 min to collect protein lysate. For immunoblotting, 10 μg of lysate from each sample was separated on 6% SDS polyacrylamide gel and electro-transferred onto PVDF membrane (Amersham Pharmacia, ON). The membrane was blocked at room temperature for 2 h with 5% skim milk, incubated with mouse anti-MLCK (K36 clone) polyclonal Ab (Sigma-Aldrich), or mouse anti-calponin antibody (Sigma-Aldrich) at room temperature for 2 h, followed by secondary antibody HRP-goat anti-mouse IgG prepared in 1% skim milk. All the blots were developed by enhanced chemiluminiscence as recommended by the supplier (Amersham Pharmacia, ON). β-actin was used as internal control. The intensity of smMLCK, myosin, calponin and β-actin bands was determined by using AlphaEase FC software version 3.1.2 relative to control loading levels. For signaling, the intensity of phosphorylated forms of ERK, JNK, P38, myosin and smMLCK was normalized with the intensity of total ERK1/2, JNK and P38, respectively.

### Syk and Lyn Knock-down in HASM Cells by Lentiviral shRNA Transduction

Lyn and Syk kinases were silenced by transducing HASM cells with pseudotyped lentiviral vector (clone Id: V2LHS-134140; V2LHS-153702**)** expressing specific Syk and Lyn shRNA, respectively (Open-Biosystems, Huntsville, AL). 293T cells used for virus production and titration, were cultured in Dulbecco’s medium (HyClone, Logan, UT) supplemented with 10% fetal bovine serum (FBS), and 1% penicillin/streptomycin/glutamate (Gibco, Grand Island, NY) as described in [19]. A control shRNA unrelated to Lyn and Syk sequence (scramble shRNA) was used as a transduction control. For knocking-down the protein expression of these kinases, HASM cells were transduced at a multiplicity of infection (MOI) of 10 in the presence of polybrene (8 μg/ml). In brief, cells were exposed to recombinant lentivirus for 2 h at 37°C, medium replaced and cultured for additional 72 h. Transduced cells were selected with puromycin. The average transduction efficiency was determined by FACS using the turbo-green fluorescent protein (tGFP). Viability of the transduced cells undergoing experiment was >98% as assessed by trypan blue dye after completion of the experiment.

### Statistical Analysis

All the data were performed from at least three experiments. Statistical analysis was performed by doing *Mann-Whitney U test* or one way ANOVA with 95% confidence level using GraphPad Prism Software Version 3.02 for Windows (GraphPad Software Software, San Diego, CA, USA). P values <0.05 were considered statistically significant.

## Results

### IgE Augments smMLCK mRNA and Protein Content in HASM Cells through FcεRI

**Figure 1:**
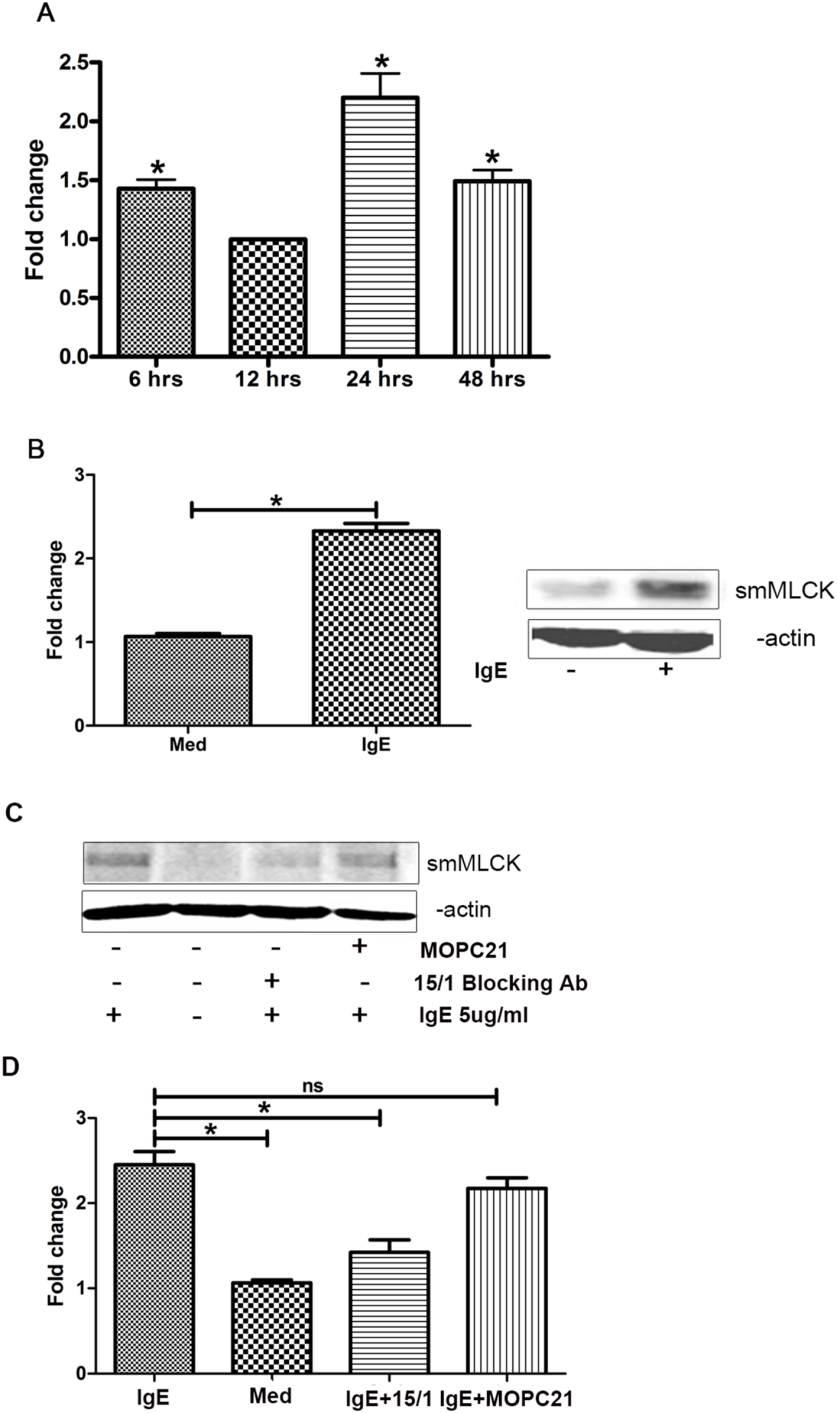
IgE enhances smMLCK expression in HASM cells through FcεRI. (**A**) HASM cells were serum-deprived for 48 h and then stimulated with IgE 5 μg/ml. Shown is fold increase in smMLCK mRNA level in IgE-stimulated HASM cells compared to unstimulated cells at corresponding time points. (**B**) HASM cells were stimulated with IgE 5 μg/ml for 48 and smMLCK protein expression was assessed by western blotting. The intensity of smMLCK band was normalized with that of β-actin. The shown blot is representative of three different experiments. (**C**) Cells were pretreated with anti-FcεRI mAb15/1 for 1 h before stimulation with IgE. MOPC21 was used as an isotype control. Western blot is a representative of three different experiments showing smMLCK protein content in different treatment groups. Fold change in the level of smMLCK upon different treatment as compared to untreated control is shown in the graphs. One way ANOVA was performed to determine the significance of data. *P*<0.05 (*), (n>3).

**Figure 2:**
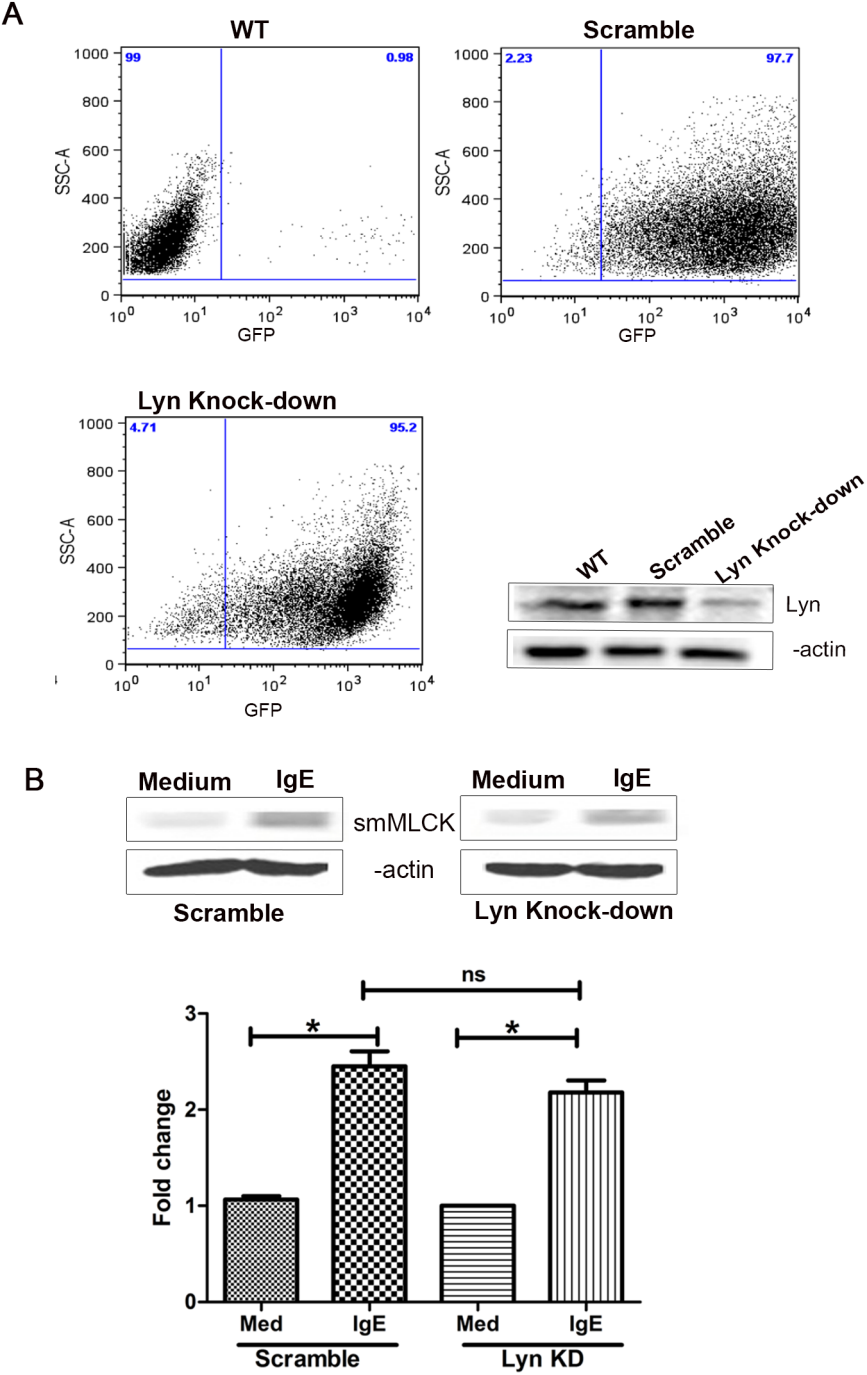
IgE-mediated smMLCK protein expression is not affected by Lyn knock-down. Lyn knock-down was induced in HASM cells by transduction with lentiviral vector expressing Lyn specific shRNA. shRNA against unrelated scramble sequence was used as control. (**A**) Lentiviral transduction efficiency was found to be more than 90% for both scramble and Lyn specific shRNA as determined by measuring GFP content. Lentivirus-induced Lyn knock-down in HASM cells was shown by western blotting. (**B**) Protein extracts were prepared from Lyn silenced and scramble HASM cells, stimulated with IgE 5 μg/ml for 48 h after serum deprivation and smMLCK protein content was assessed by western blotting (n>3). One way ANOVA was performed to determine the significance of data. *P*<0.05 (*).

Previous reports indicated that IgE-rich atopic serum induces smooth muscle contractile response [9]. Furthermore, HASM of asthmatic patients showed increased level of smMLCK expression [27]. To investigate whether IgE induced smMLCK in HASM cells, we first analyzed the expression of smMLCK in IgE-stimulated HASM cells. IgE (5 μg/ml) enhanced the smMLCK mRNA level significantly at 6, 24 and 48 h post stimulation (n>3, P<0.05) (Figure 1A). Furthermore, Western blotting experiments show that IgE (48 h) also augments the smMLCK protein expression in HASM cells (Figure 2B).

We then investigated the involvement of FcεRI and FcεRII/CD23 receptor in the regulation of smMLCK expression in HASM cells using FcεRI blocking monoclonal Ab (mAb) (15/1) directed against the IgE binding site [28], [29]. Serum-deprived HASM cells were pretreated with 10 μg/ml of mAb 15/1 or mouse IgG1 isotype control (MOPC21) for 1 h and then stimulated with IgE (5 μg/ml) for 48 h. As shown in Figure 1C–D, in contrast to isotype control mAb (MOPC21), pre-treatment with mAb15/1 significantly diminished IgE-enhanced expression of smMLCK (P<0.05, n = 3 in HASM cells. Furthermore, anti-CD23/FcεRII mAb did not affect IgE mediated smMLCK expression (Figure S2). Taken together, these results suggest that IgE stimulates the expression of smMLCK in HASM cells mainly via FcεRI.

### shRNA-mediated Syk, but not Lyn Knock-down Inhibits IgE-induced smMLCK Protein Expression in HASM Cells

**Figure 3:**
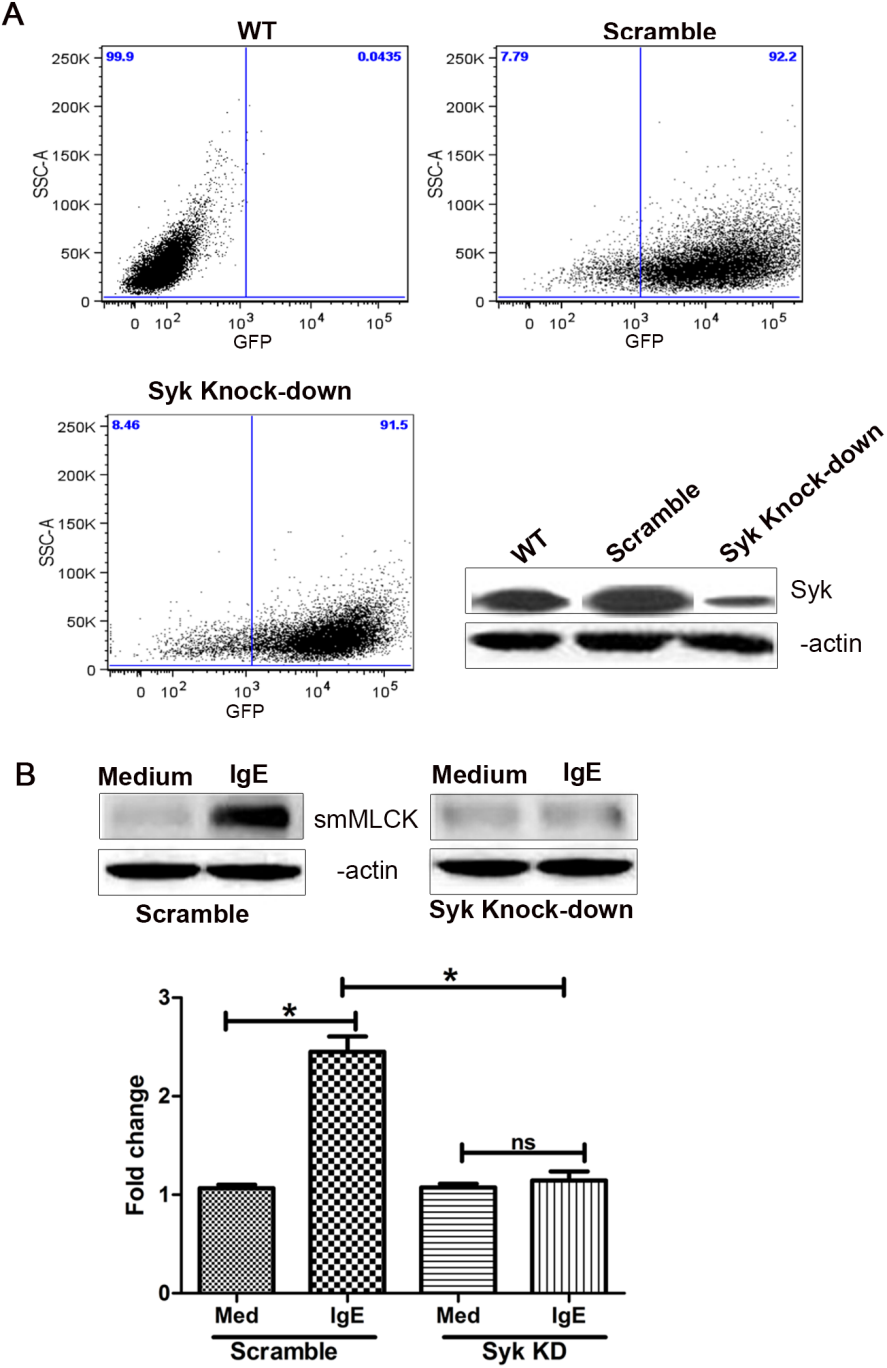
Syk knock-down abrogates IgE-mediated smMLCK protein expression. Syk knock-down was induced in HASM cells as described in Figure 2. shRNA against unrelated scramble sequence was used as control. (**A**) Transduction efficiency was analyzed by FACS using GFP as a marker. Lentivirus-transduced Syk knock-down was shown by western blotting. (**B**) Protein extracts were prepared from Syk silenced and scramble HASM cells which were stimulated with IgE 5 μg/ml for 48 h and smMLCK protein content was assessed by western blotting (n>3). One way ANOVA was performed to determine the significance of data. *P*<0.05 (*).

IgE mediates its action through the activation of FcεRI receptor followed by phosphorylation of Lyn and Syk kinase [30], [31]. We have previously shown that IgE activates FcεRI and induces the release of cytokines from HASM cells through a Syk-dependent pathway [19]. However, the role of Lyn in IgE signaling in HASM cells is completely unknown. Having identified that smMLCK protein expression is modulated by IgE through FcεRI; our next aim was to understand the role of Lyn and Syk kinase in this process. Lyn and Syk kinase knock down was performed in HASM cells by transducing HASM cells with a pseudotyped lentiviral vector expressing target-specific shRNA [19]. The transduction efficiency of ASM cells was examined by FACS analysis using the vector turboGFP (tGFP) reporter gene. As demonstrated by FACS analysis, >95% of the lentiviral transduced cells were tGFP^+^ (Figure 2A and Figure 3*A*). Transduction of cells with specific Lyn or Syk shRNA resulted in a highly significant decrease in their expression, as shown by Western blotting in figure 2A and 3A, respectively. Scramble shRNA was used as transduction control. In Syk silenced HASM cells, the effect of IgE on smMLCK protein upregulation was lost (Figure 2B). However, Lyn-silenced HASM cells responded to IgE in a similar fashion as scramble-transduced cells and no significant reduction in smMLCK expression was observed due to loss of Lyn (Figure 3B). Collectively, our data suggest that IgE enhances the smMLCK expression through Syk-dependent pathway.

### IgE Mediates smMLCK Upregulation through MAPKs (ERK1/2, P38, JNK) and PI3 Kinase

**Figure 4:**
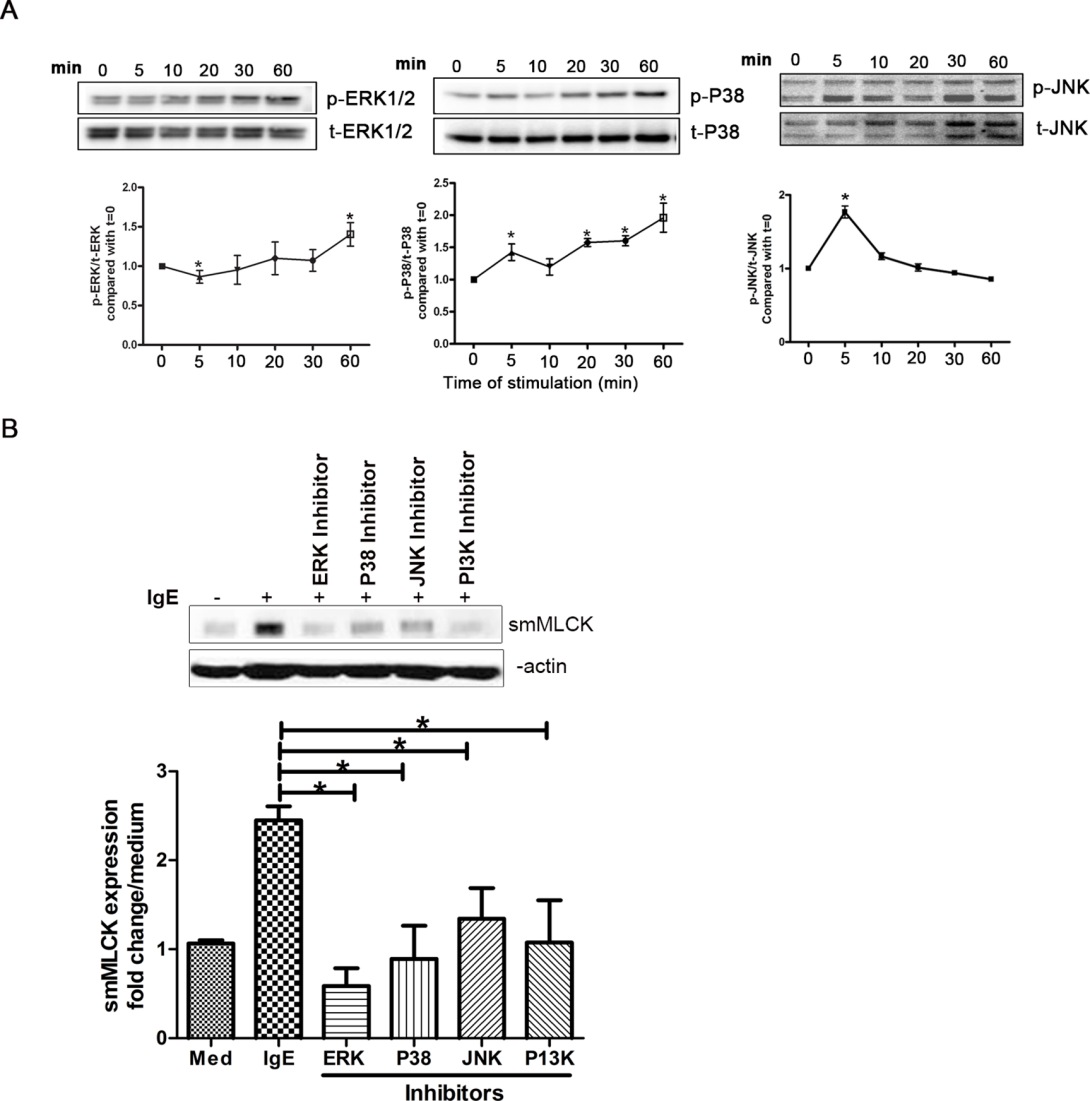
IgE mediates smMLCK upregulation through MAPK (ERK1/2, P38, JNK) and PI3 Kinase. (**A**) IgE stimulation induces the phosphorylation ERK1/2, P38 and JNK. Serum starved HASM cells were stimulated with IgE (5 μg/ml) and lysate was collected at different time points. Figure 4A shows the western blots probed with antibodies recognizing phosphorylated ERK1/2, P38 and JNK. These blots were then reprobed with total ERK1/2, P38 and JNK ab, respectively (n>3). The graphs show the densitometric ratio of phosphorylated forms to total form. (**B**) Immunoblot showing smMLCK protein expression following stimulation with IgE (5 μg/ml) and ERK1/2 Inhibitor: U-0126 (10 μM), P38 Inhibitor: SB-203580 (10 μM), JNK Inhibitor: SP600125 (40 nM) or PI3K Inhibitor Wortmanin (100 nM). Graph shows fold change in the level of smMLCK protein normalized to β-actin in different treatment groups as compared to untreated control. (n = 3). One way ANOVA was performed to determine the significance of data. *P*<0.05 (*).

We then sought to uncover the signaling mechanisms of IgE-induced smMLCK expression. IgE mediates its effect via multiple signaling pathways in inflammatory cells that include MAPK pathways in many cells including murine bone marrow-derived mast cells and B cells [31], [32]. In particular, the role of ERK1/2, P38 and JNK in IgE-induced mediators release, cell degranulation and survival is clearly established in basophils, mast cell lines, and neutrophils from asthmatic subjects [32], [33]. To test the involvement of these signaling proteins in IgE-mediated smMLCK expression, we first established whether IgE stimulation induces their phosphorylation. IgE induced an increased phosphorylation of ERK1/2, P38 and JNK in HASM cells. As shown in figure 4A, ERK and P38 phosphorylation induced by IgE increased steadily with peak levels visible by 60 minutes. Furthermore, IgE-induced JNK phosphorylation peaked early and returned to baseline levels within 20 minutes.

Next, smMLCK protein expression was assessed in cells pretreated with pharmacological inhibitors of ERK1/2 (U0126), P38 (SB203580), JNK (SP600125) and PI3 K (Wortmannin), and then stimulated with IgE for 48 h. Inhibition of MAPK (ERK1/2) and PI3K pathway resulted in a significant reduction of IgE-mediated smMLCK protein expression (P<0.05, n = 3) (Figure 4B). Pre-treatment with inhibitors of P38 and JNK also inhibited the IgE-induced smMLCK upregulation; however the effect was lesser than the inhibition of ERK1/2 and PI3K. These results suggest that ERK1/2 and PI3K, and to lesser extent P38 and JNK, are involved in IgE-mediated enhanced expression of smMLCK protein in HASM cells.

## Discussion

IgE binding to FcεRI in inflammatory cells was earlier thought to be a “passive sensitization” step requiring subsequent allergen/antigen cross-linking. Recent data suggests that IgE binding alone (sensitization) have critical effector functions on cellular activation. IgE sensitization induces pro-survival effects in mast cells, monocytes and neutrophils [32], [33], [34]. In HASM cells, IgE sensitization induces the release of CC (CCL11, CCL5) and CXC (CXCL10, CXCL8) chemokines, and the pro-allergic cytokine thymic stromal lymphopoietin (TSLP) [17], [19], [23], [35]. In this study, we showed for the first time that IgE upregulates the expression of smMLCK HASM cell mainly through binding to the FcεRI. This phenomenon involves Syk, MAPKs (ERK1/2, P38 and JNK) and PI3K signaling pathways. Taken together, our data suggest a plausible role of IgE in modulating contractile machinery of HASM cells.

IgE-induced upregulation of smMLCK was found to involve FcεRI activation as an FcεRI-α chain-specific blocking antibody mAb 15/1, but not anti CD23/FcεRII mAb, significantly inhibited the IgE-mediated upregulation of smMLCK expression in HASM cells. The mAb 15/1 is an anti- human FcεRI alpha chain mouse antibody which inhibits the binding of IgE to membrane bound FcεRI [28], [36]. Based on our data, we suggest that IgE mediated smMLCK expression is primarily mediated through FcεRI since blocking CD23/FcεRII has no effect on this event. Notably, FcεRI is the central mediator of allergic inflammatory response and forms the basis of immediate hypersensitivity. FcεRI activation on inflammatory cells leads to a plethora of biological functions including cell degranulation, survival, and migration which could affect the outcome of allergic response. Reduction in surface FcεRI expression by means of controlling serum IgE in allergic asthmatic subjects leads to improved asthma control [37]. Our current data suggest that targeting FcεRI on HASM cells in allergic asthma may provide novel means of controlling the bronchomotor tone, in addition to the previously characterized proinflammatory cytokine and chemokine release [35].

IgE-antigen complex aggregates and activates the FcεRI which induces Lyn kinase phosphorylation. Lyn then phosphorylates immunoreceptor tyrosine activating motif (ITAM) on FcεRI providing a docking site for the activation of Syk kinase. These events subsequently result in phosphatidylinositol-3-phosphate (PIP3) production, activation of phospholipase C and thus increase in cytosolic Ca^2+^ levels [38]. In our study, we found that knock-down of Syk, but not Lyn, abrogated the IgE-mediated smMLCK protein expression in HASM cells. Early studies have indicated that Lyn is a positive regulator of FcεRI activation [38]. However, results from electron microscopy and biochemical investigations indicated that Lyn dissociates from FcεRI as soon as the later is stimulated [39], [40]. Furthermore, Lyn-associated FcεRI was reported to be less competent for signaling [40]. Emerged from here was the viewpoint that Lyn might be a negative regulator of FcεRI signaling. Moreover, Lyn^−/−^ mice displays increased serum IgE levels and increased expression of surface FcεRI on mast cells *in vivo* that account for the exacerbated allergic phenotype as compared to their WT littermates [41]. Furthermore, FcεRI -mediated activation of Lyn^−/−^ BMMCs results in greater increase in mRNAs encoding Th2 cytokines and chemokines [41], [42], [43]. In our report, knock-down of Lyn kinase could not affect IgE-mediated smMLCK protein expression, neither positively or negatively. In contrast to Lyn −/− BMMC, Lyn knock-down could not abrogate IgE-mediated IL-8 production in HASM cells (data not shown). Finally, a plausible role of other kinases of Src family such as Fyn may not be denied [44].

MAPKs play a crucial role in modulating the cellular functions such as cell proliferation, survival, muscle contraction and cell migration [45], [46], [47], [48]. ERK1/2 is reported to be essential for MLCK expression and activity in vascular smooth muscle cells and breast cancer cells [47], [49], [50], [51]; while in astrocytes, P38 but not ERK1/2 or JNK upregulates the expression of MLCK [52]. Previously, the effect of cross-linked IgE on MAPK phosphorylation of has been shown in various cell types including human intestinal epithelial cells [53], human mast cells [54], murine mast cells [55], and human basophils [56]. However, information on monomeric IgE-mediated regulation of MAPK phosphorylation is limited. *Sly et al*. [57] tested five different monomeric IgEs and demonstrated a prolonged phosphorylation of ERK with peak levels at 60 min in murine BMMCs. This event was found to be critical for cytokine production. Another report by *Kalesnikoff et al* [32] showed monomeric IgE-induced phosphorylation of P38 and JNK in murine BMMCs which peaked at 5 and 15 min, respectively. Altogether, it appears that IgE alone has the ability of inducing the phosphorylation of ERK, P38 and JNK in many human cells including HASM cells. However, whether the strength and the duration of phosphorylation is species, cell type and IgE clone-specific is unknown.

Prevalent notion suggests that smMLCK is activated by the calcium-calmodulin complex leading to myosin RLC phosphorylation, which induces contraction in HASM cells [11]. The phosphorylated form (p-serine 1760) of smMLCK is known as the inactive form. Serine 1760 is located in the calmodulin binding site in the C-terminal domain of smMLCK. Phosphorylation at this site prevents calcium-calmodulin complex from interacting with smMLCK and activating smMLCK [58]. Whether IgE regulates the activation of smMLCK and subsequent phosphorylation of myosin RLC and contributes directly in HASM cells contraction, would be interesting to investigate.

Collectively, our data supports the concept that IgE induced the expression of smMCLK in HASM cells via Syk, MAPK and PI3K signaling pathways suggesting a plausible role of IgE in modulating HASM cell contraction.

## Supporting Information

Figure S1 **Cell cycle analysis (A) and calponin expression (B) in HASM cells upon serum deprivation.**
(TIF)

Figure S2 **FcεRII/CD23 blocking mAb fails to inhbit IgE mediated smMLCK expression in HASM cells.** Cells were pretreated with anti-FcεRII/CD23 mAb (Clone M-L233) for 1 h before stimulation with IgE. MOPC21 was used as an isotype control. Western blot is a representative of three different experiments showing smMLCK protein content in different treatment groups. Fold change in the level of smMLCK upon different treatment as compared to untreated control is shown in the graphs. One way ANOVA was performed to determine the significance of data. *P*<0.05 (*), (n = 4).
(TIF)
