# Supplementary figures and images for: IgE Regulates the Expression of smMLCK in Human Airway Smooth Muscle Cells
Source: PLoS One. 2014 Apr 10;9(4):e93946. doi: 10.1371/journal.pone.0093946 (PMC3983085; doi:10.1371/journal.pone.0093946)

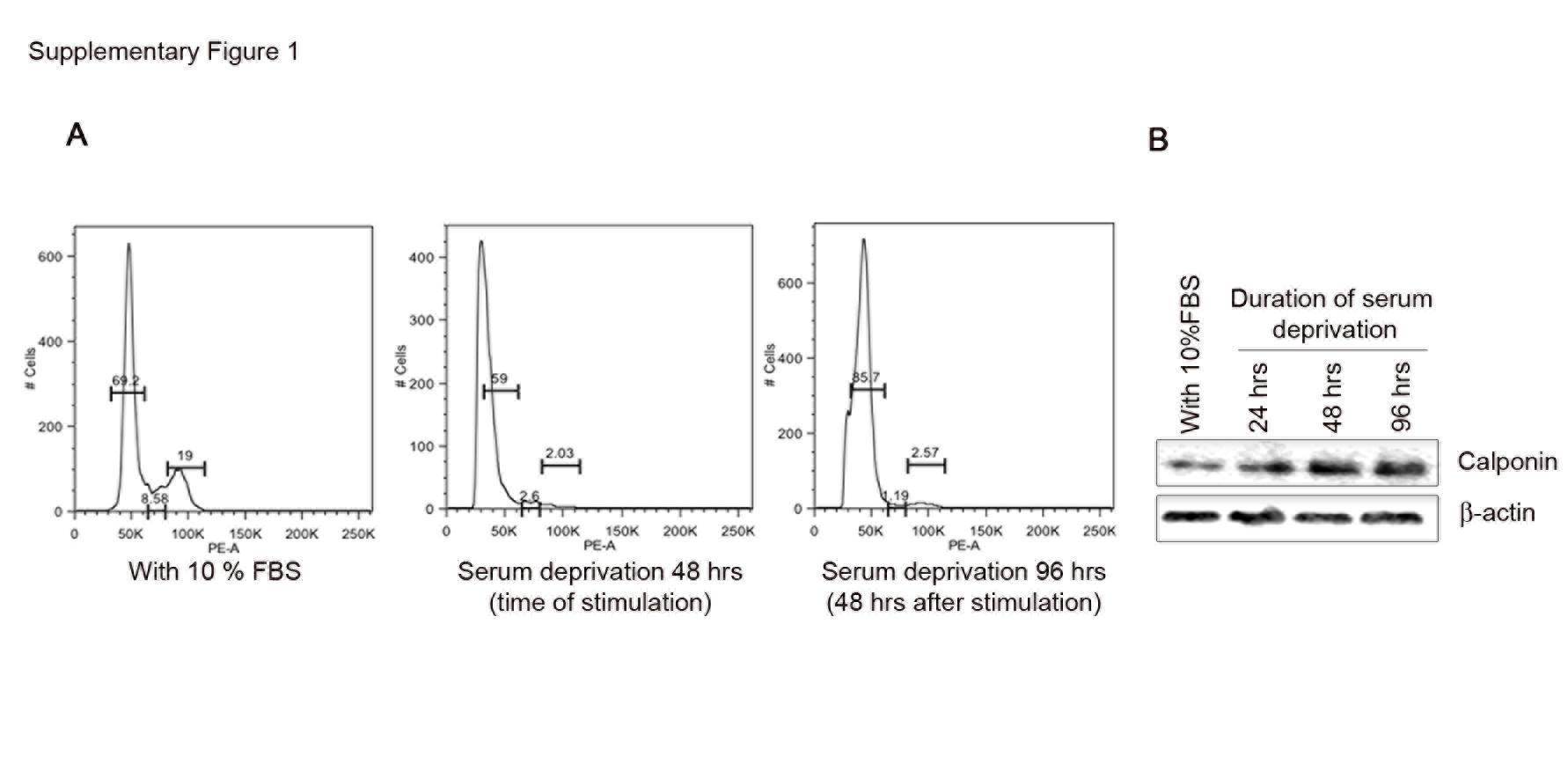

Supplement: Figure S1 — Cell cycle analysis (A) and calponin expression (B) in HASM cells upon serum deprivation. (TIF) [file pone.0093946.s001.tif]

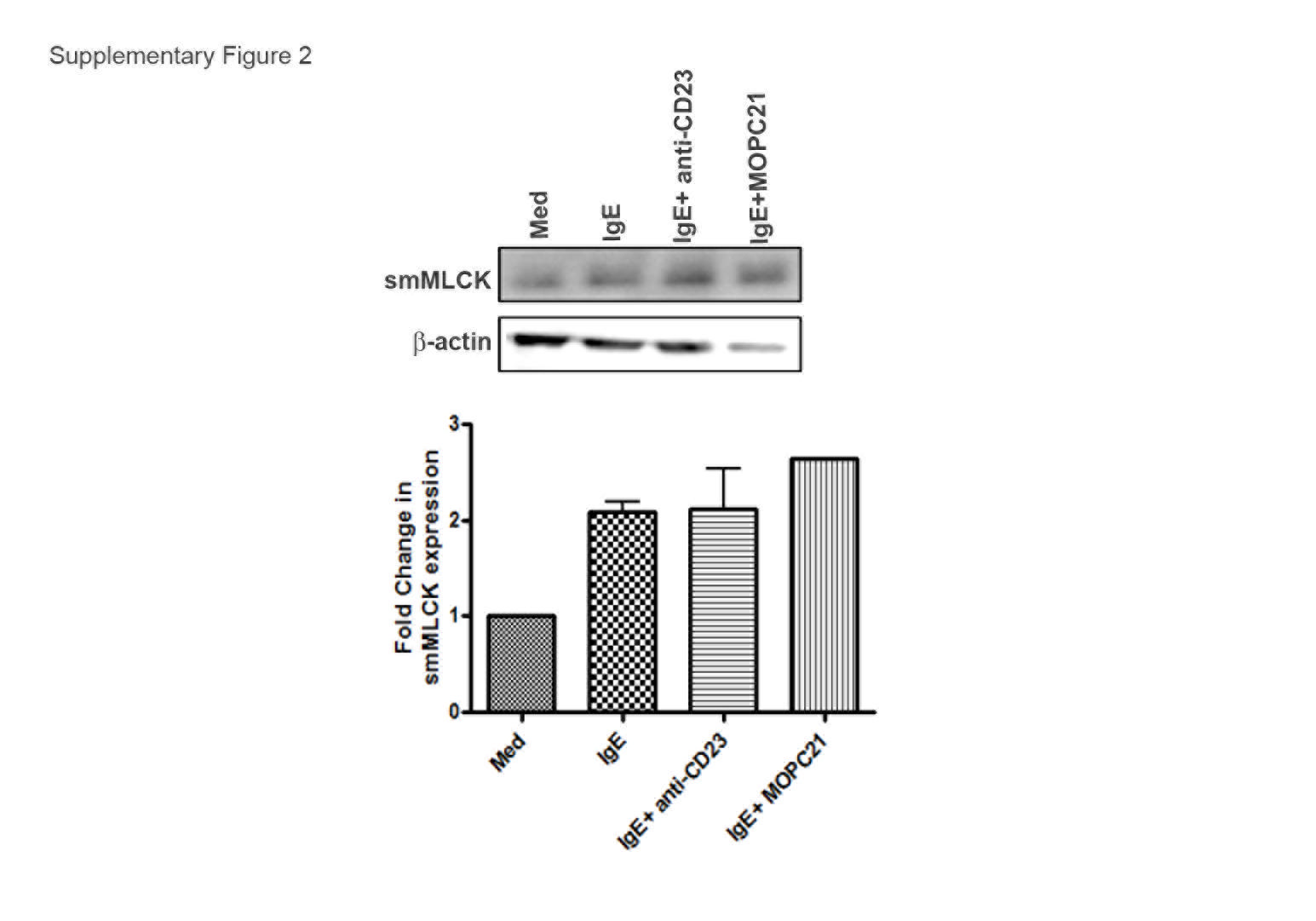

Supplement: Figure S2 — FcεRII/CD23 blocking mAb fails to inhbit IgE mediated smMLCK expression in HASM cells. Cells were pretreated with anti-FcεRII/CD23 mAb (Clone M-L233) for 1 h before stimulation with IgE. MOPC21 was used as an isotype control. Western blot is a representative of three different experiments showing smMLCK protein content in different treatment groups. Fold change in the level of smMLCK upon different treatment as compared to untreated control is shown in the graphs. One way ANOVA was performed to determine the significance of data. P<0.05 (*), (n = 4). (TIF) [file pone.0093946.s002.tif]
